# Gallbladder wall perforation secondary to empyema in Mirizzi’s syndrome: An unseen complication. A case report

**DOI:** 10.1016/j.ijscr.2020.06.040

**Published:** 2020-06-15

**Authors:** Awadh Alqahtani, Qurrat Al Ain Atif

**Affiliations:** a FRCSC(Surgical Oncology), Consultant Laparoscopic and Bariatric Surgery, King Saud Medical University, Riyadh, Saudi Arabia; b MRCS(Eng), FCPS(Pak), Dip. Laparoscopic Surgery (France), General Surgery Specialist, Dr. Sulaiman Al Habib Medical Group, Riyadh, Saudi Arabia

**Keywords:** Mirizzi’s syndrome, *Gallbladder empyema*, *Cholecystolithiasis*, *Choledocho-colic fistula*

## Abstract

**Introduction:**

Mirizzi’s syndrome is a rare but potential complication of long standing gallstone disease, particularly cholecystolithiasis.

**Case presentation:**

Being described is a case of a 42 year old patient who presented with right upper quadrant pain. Initially sent home as the pain settled but was diagnosed as Mirrizi’s syndrome on readmission and was managed accordingly. This case report is being presented with the aim of highlighting the importance of Mirizzi’s syndrome and the potential consequences it may bear.

**Discussion:**

Due to lack of symptom specificity, this entity remains undiagnosed preoperatively in majority of the cases. Although safe in expert hands, minimally invasive approach (in selected types) can be challenging and bears the risk of bile duct injuries, with a high probability of conversion.

**Conclusion:**

Delays in targeted management may lead to dire consequences ranging from empyema gallbladder to gangrene of gall bladder wall, perforation and frank sepsis. A thorough clinical history and physical examination and assessment in light of clinical investigations can lead to a timely diagnosis and management.

## Highlights

• Mirizzi syndrome is a rare entity.
• Diagnosis can be overlooked due to lack of specific signs and symptoms.
• MRCP can be useful in diagnosing while ERCP can be diagnostic as well as an interim therapeutic strategy.
• Can be potentially life threatening, if left untreated, in the settings of gallbladder empyema and perforation.
• Treatment is cholecystectomy, laparoscopic vs open, depending upon surgeon’s preference and experience.

## 1 Introduction

Mirizzi’s syndrome is a complication of chronic gallstone disease, named after the Argentinian surgeon, Pablo Louis Mirizzi’s, who originally described the condition in 1948 [[1], [2], [3]]. The incidence varies from 0.05 to 2.7% of patients with cholelithiasis [4,5], 0.06–5.7% of patients undergoing cholecystectomies and 1.07% of patients undergoing endoscopic retrograde cholangiopancreatiography (ERCP) [6].

A stone impacted in the Hartman’s pouch or cystic duct causing extrinsic compression of the bile ducts is implied as underlying mechanism [2,4,[7], [8], [9]]. Constant pressure, chronic inflammation, ulceration and superadded infection lead to drastic surgical emergencies namely, cholecystobiliary or cholecystoenteric fistulae, empyema gallbladder, gangrene or perforation of gallbladder and sepsis [9,10].

Various anatomical anomalies are identified as predisposing factors to the development of Mirizi’s syndrome [10]. Beltran described nine variants associated with Mirizzi’s syndrome [2,6]; (i) atrophic gallbladder with either a thick or a thin wall; (ii) obliterated cystic duct; (iii) long, low-inserting cystic duct; (iv) normal, short cystic duct; (v) partial compression of bile duct by a gallbladder stone or stone eroding into bile duct; (vi) normal caliber distal bile duct; (vii) dilated proximal bile duct; (viii) anomalous communication between bile duct and gallbladder; and (xi) anomalous communication between gallbladder and stomach, duodenum, colon or other abdominal viscera [6].

Over the course of years, various classification systems have been proposed for this syndrome. All types clinically represent evolving stages in disease progression [2]. Csendes classification is a rather simple way which classifies Mirizzi’s syndrome into 4 types [3,10] (Table 1).

**Table 1:** Csendes classification.

Type I	Extrinsic compression of the common duct due to an impacted stone at gallbladder neck or cystic duct
Type II	Cholecystobiliary (either cholecystohepatic or cholecysto-choledochal) fistula with defect less than 1/3 of the duct circumference
Type III	Fistula formation, wall defect up to 2/3
Type IV	Fistula formation, completed destruction of the duct wall

Modified version of the classification systems have recognized cholecystoenteric fistula as Type V.

Mirizzi’s syndrome has a female preponderance with an age range of 53–70 years [2,10]. Chronic gallstone disease is associated with this condition with an average disease period of 29.6 years [10].

There are 2 forms of the disease; acute and chronic. Acute form is most commonly encountered [10]. Lack of specific signs and symptoms make it difficult to diagnose early with a pre-operative diagnosis rate ranging from 8 to 62.5% [2,10]. Clinical presentation ranges from asymptomatic to nonspecific symptoms as obstructive jaundice (27.8–100%), pain right upper abdominal quadrant (16.7–100%) and fever [6]. On rare occasions, it may present as gallstone ileus [6].

Diagnosis is based on a combination of blood workup and radiological investigations. Elevated liver enzymes with hyperbilirubinemia and elevated aminotransaminases along with leukocytosis are commonly found [10]. Rarely, CA19-9 may be markedly raised, leading to a suspicion of gallbladder or biliary tract carcinoma [10]. A 5 times increased rate of gallbladder malignancy was related to Mirizzi’s syndrome in some studies [1]. 6–28% of patients have been reported to have a cholangiocarcinoma, by some [10].

Ultrasonography may show gallstones and intrahepatic biliary dilatation. Sensitivity ranges from 8.3 to 27% [10]. Computerized tomography (CT) scan is ideally used to rule out malignancy. Magnetic resonance cholangiopancreatography (MRCP) has a diagnostic accuracy of 50% [11]. It can show features typical of Mirizzi’s syndrome and can rule out a fistula. Endoscopic retrograde cholangiopancreatography (ERCP) is diagnostic as well as therapeutic, with a diagnostic accuracy ranging from 55% to 90% [11]. More than 50% patients are diagnosed intraoperatively [11].

Treatment of Mirizzi’s syndrome is surgical, tailored according to the type, patient condition and surgeon’s experience. Cholecystectomy is performed in all settings, usually staged in type II.

Laparoscopic approach in a known case of Mirizzi’s syndrome can be disastrous as it may lead to bile duct injuries because of distorted anatomy and severe inflammation [1,10]. This approach is associated with a high conversion rate [6].

ERCP remains gold standard for identifying Mirizzi’s syndrome [1,4]. It may serve as a bridge to surgery in settings of cholangitis [2,4], since stent insertion may alleviate jaundice and stone extraction can be performed concomitantly. It is also the procedure of choice in poor surgical candidates.

The following case is being presented in line with SCARE criteria [12].

## 2 Case presentation

A 42 year old male patient presented to the emergency department of our hospital with right upper abdominal quadrant pain associated with nausea of 2 days’ duration. Patient had no significant medical history or past surgical history. He had no known allergies and was not a smoker. Patient was hemodynamically stable, blood workup did not reveal any abnormality.

Ultrasonography showed single gallstone with normal gallbladder wall thickness and no pericholecystic fluid. Common bile duct was found to be normal.

The patient’s condition improved upon supportive treatment and hence he was sent home with a diagnosis of biliary colic, settled with medication.

Three days later, the patient presented to the outpatient clinic. Patient was hemodynamically stable, icteric with a tender right upper abdominal quadrant and positive Murphy’s sign.

Hematology workup revealed a white cell count of 15.48 × 10^3^/mm^3^, bilirubin of 108 mg/dl, alkaline phosphatase of 334 IU/l and gamma glutamyl transferase of 733 IU/l.

**Fig. 1:**
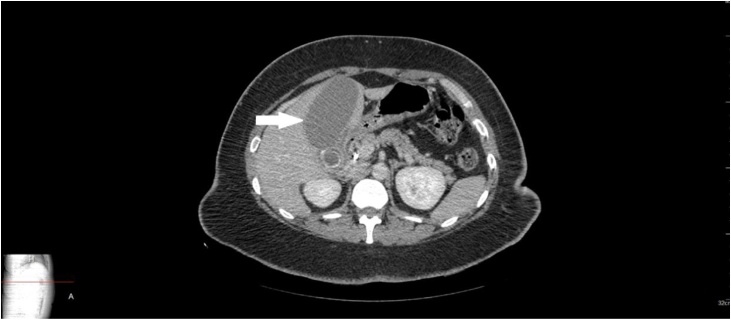
CT scan showing distended gallbladder with an impacted stone in neck/cystic duct.

**Fig. 2:**
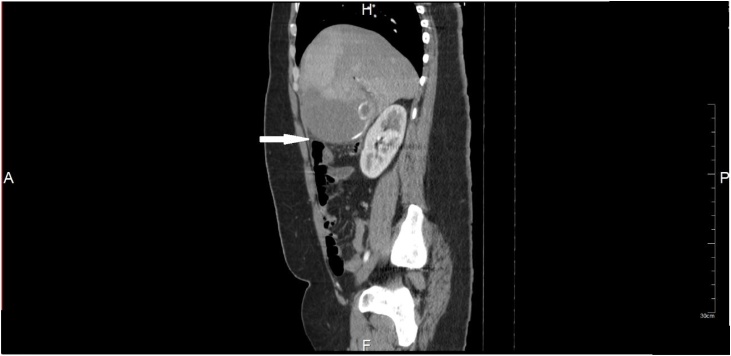
Sagittal view of CT scan.

**Fig. 3:**
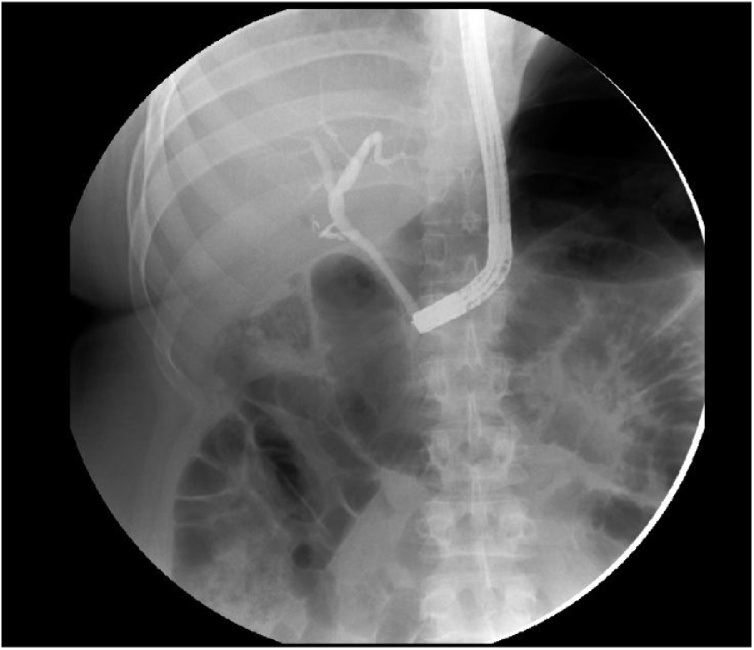
ERCP unable to visualize cystic duct.

CT scan abdomen and pelvis showed a distended gallbladder with a large stone in neck/cystic duct (Fig. 1, Fig. 2). ERCP was performed the next day as an interim procedure to bring the bilirubin down and improve the patient’s condition before embarking on cholecystectomy, sphincterotomy was done and stent was placed. Large cystic duct stone was seen which could not be retrieved (Fig. 3).

**Fig. 4:**
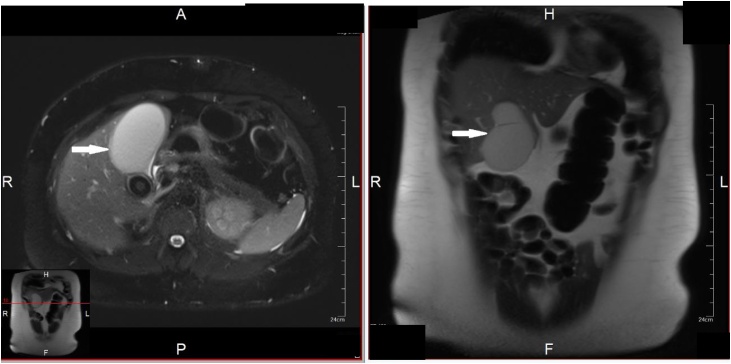
MRCP showing Mirizzi type I.

Liver functions and patient’s condition failed to improve. MRCP was done which showed Mirizzi’s syndrome type I based on the finding of significantly distended gallbladder due to impacted 3.2 × 2.2 cm stone in gallbladder neck/cystic duct with extrinsic compression of common hepatic duct (Fig. 4).

**Fig. 5:**
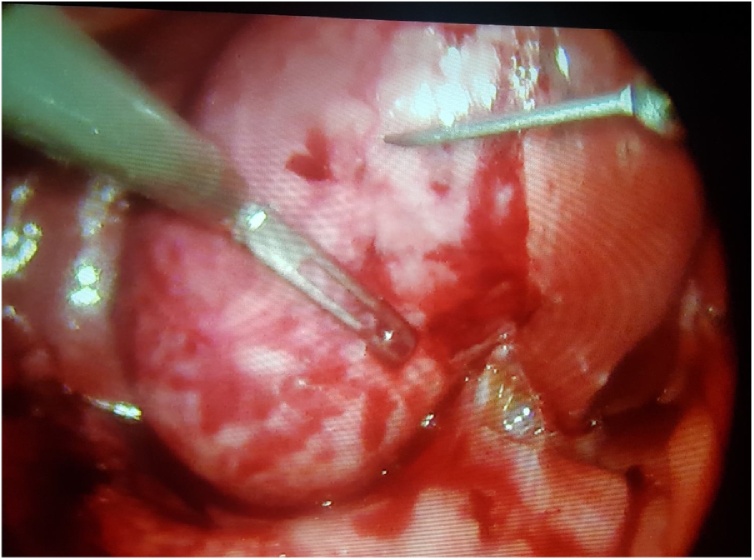
Empyema gallbladder, per-operatively.

**Fig. 6:**
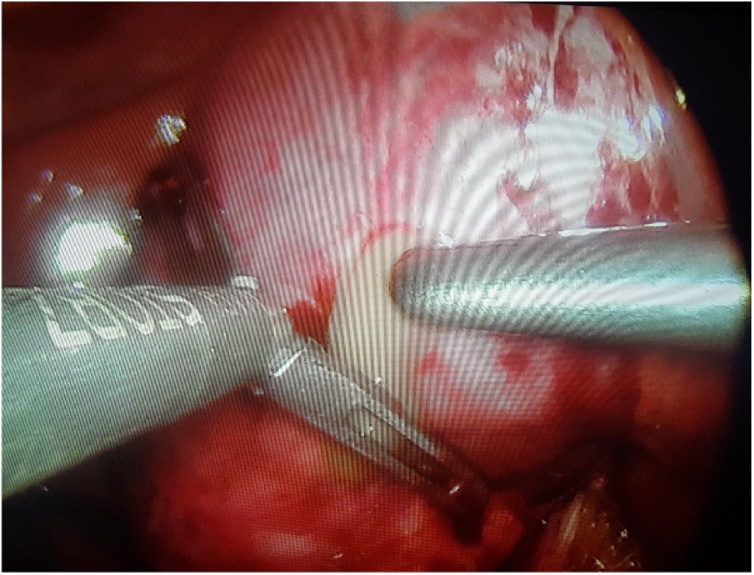
Aspiration of pus from gallbladder.

**Fig. 7:**
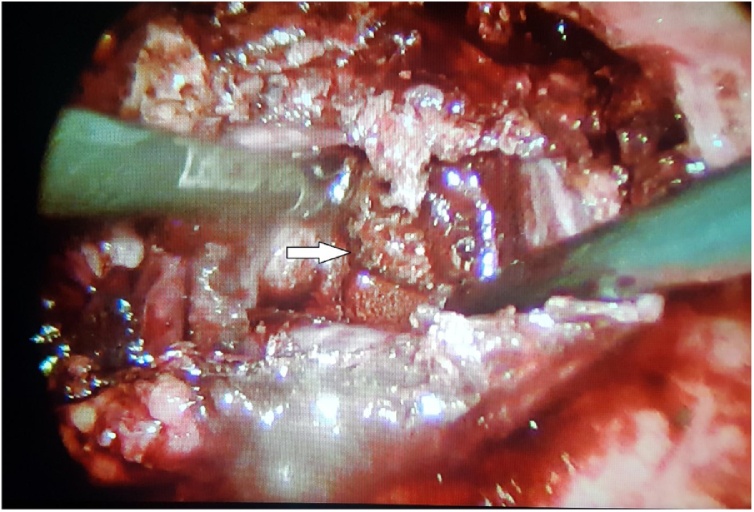
Stone impacted at neck of gallbladder (white arrow).

Patient was taken to operating room for cholecystectomy but preoperatively a huge empyema (Fig. 5) was encountered with a necrotic posterior gallbladder wall. 30cc of pus was aspirated from the gallbladder (Fig. 6). A large stone impacted in the neck of gallbladder was located and extracted (Fig. 7). Furthermore, necrotic posterior wall of gallbladder with a small perforation were identified. Cholecystectomy was done and patient was shifted to recovery room. The postoperative period was uneventful with normalization of liver function tests. Patient was discharged home on day 2 in stable condition.

## 3 Discussion

Mirizzi’s syndrome is a diagnostic challenge [10] due to nonspecific clinical presentation. Although rare, it has a female preponderance and can occur in any age group [2,10].

In our case, the patient presented to emergency but the diagnosis was missed due to non-specific symptoms. Later, he presented as a case of cholangitis and was diagnosed to have Mirizzi’s syndrome type I based on MRCP. Initially ERCP and stenting was done to alleviate symptoms. But in the meanwhile, patient’s symptoms worsened and he was taken for surgery whereby an empyema with a necrotic gallbladder were discovered.

Such complication with Mirizzi’s syndrome has not been reported in the literature before.

Importance of reporting this case lies in the fact that being rare, Mirizzi’s syndrome can be overlooked by physicians and this can lead to dire consequences as in our case where the patient ended up having empyema gallbladder with necrotic gallbladder wall with a localized perforation. Further delay could have landed patient in biliary peritonitis and sepsis. Ultrasound can be highly operator-dependent, so ERCP or MRCP should be performed to confirm diagnosis.

Although laparoscopic approach to this condition is controversial [1,6,10] but in experienced hands, it is usually safe and bears the advantages of short hospital stay, quicker recovery, lesser postoperative pain. So we decided to approach the gallbladder laparoscopically and after aspirating empyema, gallbladder wall was opened up, stone extracted and cholecystectomy performed.

A thorough clinical history and physical examination and assessment in light of clinical investigations can lead to a timely diagnosis and prevent bile duct injuries preoperatively. Surgical management is tailored to the type of syndrome, patient’s condition and surgical expertise [10].

## Sources of funding for your research

None.

## Ethical approval

A case report.

## Consent

Written informed consent was obtained from the patient for publication of this case report and accompanying images. A copy of the written consent is available for review by the Editor-in-Chief of this journal on request.

## Author contribution

1. Dr. Awadh Roban Alqahtani, performed the surgery, conceived the idea.
2. Dr. Qurrat Al Ain Atif, assisted the surgery, performed literature search and wrote the manuscript.

## Registration of research studies

None.

## Guarantor

Dr. Awadh Roban Alqahtani.

## Provenance and peer review

Not commissioned, externally peer-reviewed.

## Declaration of Competing Interest

None.
